# Plasma and Milk Variables Classify Diet, Dry Period Length, and Lactation Week of Dairy Cows Using a Machine Learning Approach

**DOI:** 10.3390/metabo15110698

**Published:** 2025-10-28

**Authors:** Xiaodan Wang, Sanjeevan Jahagirdar, Bas Kemp, Josef J. Gross, Rupert M. Bruckmaier, Edoardo Saccenti, Ariette van Knegsel

**Affiliations:** 1 Adaptation Physiology Group, Department of Animal Sciences, Wageningen University & Research, 6708 WE Wageningen, The Netherlands; wangxiaodan@caas.cn (X.W.); bas.kemp@wur.nl (B.K.); 2 Laboratory of Systems and Synthetic Biology, Wageningen University & Research, 6708 WE Wageningen, The Netherlands; jahagirdar.sanjeevan@gmail.com; 3 Institute of Food Science and Technology, Chinese Academy of Agricultural Sciences, Beijing 100193, China; 4 Veterinary Physiology, Vetsuisse Faculty, University of Bern, 3012 Bern, Switzerland; josef.gross@unibe.ch (J.J.G.); rupert.bruckmaier@unibe.ch (R.M.B.)

**Keywords:** cattle, algorithm, transition period, cow management, metabolism

## Abstract

**Background/Objectives**: The aim of this study was to classify cows with respect to different diets, dry period (DP) lengths, and lactation weeks based on body weight, milk variables, and plasma metabolites measured in early lactation. **Methods**: Holstein–Friesian cows (*n* = 95) were randomly assigned to three DP lengths (0, 30, or 60 d; *n* = 31, 34, and 30) and two early-lactation diets (lipogenic: *n* = 47; glucogenic: *n* = 48) in a 3 × 2 factorial design. From 10 d pre-calving to 8 weeks postpartum, cows received experimental diets. An XGBoost model was trained for classification using weekly body weight, milk variables, and plasma metabolites, validated via 1000 repeated hold-out partitions with stratified sampling. **Results**: Classification performance for lactation week, relative to week 1 in lactation, was good, with an area under the curve (AUC) > 0.9, independent of diet or DP length. The classification for 0 d vs. 60 d DP length was better than that for 0 d vs. 30 d or 30 d vs. 60 d DP length, showing an AUC > 0.8, independent of diet or lactation week. The top features to classify diet were plasma urea and milk fat content. Milk yield and protein content were the important features for classifying lactation weeks regardless of diet, while milk fat content was a critical predictor specific to the glucogenic diet. **Conclusions**: Our findings demonstrate that milk and plasma features can retrospectively classify management groups in early lactation using machine learning approaches.

## 1. Introduction

During early lactation, increased feed intake cannot meet the cows’ rapidly increasing energy requirements for milk production [1]. To compensate for this energy deficit, cows mobilize body fat [2]. The increased energy demand and body fat mobilization are typical characteristics of the high metabolic load that cows experience during the early–lactation period [3,4]. Moreover, this high metabolic load is associated with decreased immune competence [5], the occurrence of ketosis [6,7], and an increased risk for diseases and disorders [8].

Dietary strategies can modulate metabolic status. Several studies have investigated the effects of different diets and dry period (DP)lengths on the metabolic status and milk production of dairy cows in early lactation [9,10,11]. Feeding a more glucogenic diet during early lactation reduced plasma non-esterified fatty acids (NEFA) and β-hydroxybutyric acid (BHB) concentrations [12]. A more lipogenic diet, provided as unsaturated fat in rumen-protected form, increased milk production without affecting the body condition of the cows [13]. Dietary supplementation with cracked rapeseed or rumen-protected vegetable fat increased milk energy production [14]. Furthermore, dietary supplementation with C16:0 reduced plasma insulin concentration, increased plasma NEFA, and intensified the negative energy balance (NEB) in early-lactation cows [15]. Similarly, shortening or eliminating the DP can reduce metabolic stress [16] but may reduce subsequent milk yield [17,18]. The above studies highlight the delicate balance between dairy cow management strategies, productivity, and health.

Previous studies focused on comparing the differences between groups using traditional statistical methods without considering the overall data profile. Complementarily, machine learning methods have been applied to classify cows based on metabolic status [19,20] and also retrospectively assess if cows were fed a total mixed ration or a pasture-based ration [21].

Although machine learning methods seem to have potential, there is still limited evidence on how well they can capture management-related variables in dairy production [22,23]. As a machine learning algorithm, XGBoost has several advantages and we have previously used with success [24]: (1) Its tree-structured algorithms naturally support feature selection, automatically identifying the most predictive biomarkers; (2) The gradient boosting mechanism accurately models complex nonlinear patterns, significantly improving prediction accuracy; (3) It provides feature importance rankings, providing an interpretable path to understanding biological mechanisms. These features enable XGBoost to not only break through the bottlenecks of traditional methods but also provide biologists with analytical tools that have both predictive performance and mechanism exploration value. Therefore, this study used the XGBoost machine learning algorithm to classify dairy cows according to diet, DP length, and lactation week by analyzing body weight, plasma metabolites, and milk composition variables. Ultimately, this classification offers producers, veterinarians, or farm advisors a novel tool to identify underlying causes of health issues and to make timely management adjustments aimed at improving the metabolic health of cows. By integrating multidimensional biological indicators with advanced machine learning, this work provides a more precise and practical method for individualized metabolic monitoring in dairy herds, addressing a critical gap in current metabolic management strategies.

## 2. Materials and Methods

**Figure 1:**
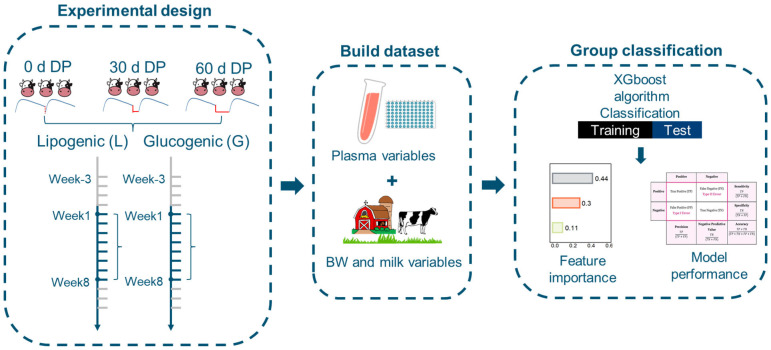
Overview of experimental design and data processing. (1) Plasma and milk samples and BW data were obtained from week 1 to week 8 relative to calving from cows with a 0 d, 30 d, or 60 d DP and fed either a glucogenic or a lipogenic diet. (2) Plasma and milk variables were analyzed. Plasma and milk variables, BW data, and cow characteristics were used to build the dataset. (3) Cows were classified with respect to diet, DP length, or lactation week using plasma and milk variables and BW through the XGBoost algorithm, including determination of feature importance and model performance. The model performance table was obtained from [27]. Abbreviations: BW = body weight; DP = dry period.

The Institutional Animal Care and Use Committee of Wageningen University (Wageningen, The Netherlands) approved the experimental protocol (registration number 2010026). The experimental design, DP lengths, and diets have been described earlier [11]. In short, Holstein–Friesian cows (*n* = 95) were selected from the Dairy Campus research herd (Wageningen Livestock Research, Lelystad, The Netherlands) and were blocked by parity (2nd, 3rd, or ≥4th parity), expected calving date, milk yield in the previous lactation, and body condition score. Within blocks of 6 cows, cows were randomly assigned to DP length (*n* = 31, *n* = 34, and *n* = 30 for 0, 30, and 60 d DP, respectively) and early-lactation diet (*n* = 47 and *n* = 48 for lipogenic and glucogenic, respectively) (Figure 1). Diets were equal in net energy content [25] and equal in intestinal digestible protein and degraded protein balance [26]. Before calving, lactating cows were fed a lactation diet supporting 25 kg of milk. All cows were fed 1 kg/d of glucogenic or lipogenic concentrate from 10 d before the expected calving date. At calving, the supply of concentrates was increased stepwise by 0.5 kg/d until it reached 8.5 kg/d. The main ingredient in the glucogenic concentrate was corn, while the main ingredients in the lipogenic concentrate were sugar beet pulp, palm kernel, and rumen-protected palm oil. Forage was offered ad libitum throughout this study and was composed of grass silage, corn silage, wheat straw, and rapeseed meal or soybean meal (51:34:2:13, dry matter basis) (Table 1). Cows were housed in a freestall with a slatted floor and cubicles and were milked twice daily (0500 and 1630 h).

### 2.1. Body Weight (BW), Milk Yield, and Composition

After calving, BW was recorded after each milking and averaged per week. Milk yield for each cow was recorded daily and averaged per week. Milk samples for the analysis of fat, protein, and lactose percentage, urea concentration, and somatic cell count (**SCC**) (ISO 9622; Qlip NV, Zutphen, The Netherlands) were collected 4 times per week (Tuesday afternoon, Wednesday morning, Thursday afternoon, and Friday morning) from week 1 to week 8 in lactation. Fat- and protein-corrected milk (FPCM) was calculated per week as follows [28]:

$$FPCM\left(\mathrm{kg}\right)=milk\left(\mathrm{kg}\right)\times (0.337+0.116\times fat\left(\%\right)+0.06\times protein\left(\%\right))$$

### 2.2. Blood Sampling and Analysis

Blood sampling was described earlier [16]. In plasma, concentrations of glucose and urea were measured using commercial kits no. 61269 and no. 61974 (BioMérieux, Marcy l’Etoile, France), as previously described [29]. Concentrations of NEFA and BHB were enzymatically measured using kit no. 994-75409 (Wako Chemicals, Neuss, Germany) and kit no. RB1007 (Randox Laboratories, Ibach, Switzerland), as previously described [29]. Insulin-like growth factor-1 (IGF-1) and insulin were measured using radioimmunoassay, as previously described [30].

### 2.3. Data Imputation and Processing

Data on BW and milk yield from week 1 to week 8 were completely available. Milk fat percentage, milk protein percentage, milk lactose percentage, milk SCC (*1000 cells/mL), FPCM (kg/d), NEFA (mmol/L), BHB (mmol/L), glucose (mmol/L), urea (mmol/L), IGF-1 (ng/mL), and insulin (μIU/mL) had missing values (<40% per cow per variable). Data are missing completely at random. A random forest algorithm was used to impute the missing data using the R (version 4.2.1) with “*missForest*” package (version 1.5) [31]. The random forest model was constructed with default parameters of 100 trees and iterated 10 times.

### 2.4. Classification of Diet, Dry Period Length, and Lactation Week

The XGBoost algorithm [32] was used to classify dairy cows into diet, DP length, and lactation week. Stratified sampling was used to divide the data into a training set (containing 80% of the data) and a test set (containing 20% of the data) for model building and internal validation. The XGBoost model was trained to perform predictions on differentiating between 2 classes within a variable. The models were built with a maximum decision tree depth of 4 splits and a learning rate of 0.1. Since the number of cows in each group was exactly the same for all classifications, we did not add any additional weighing to the samples and kept the weight of all samples the same. Analysis: The area under the curve (AUC) of the receiver operating characteristic (ROC) was calculated to evaluate model performance. In order to prevent overfitting, with the addition of every decision tree in the model, we calculate the AUC of the model on both the training and test datasets. The model stopped adding decision trees to the ensemble as soon as the model’s performance on the test dataset decreased, irrespective of the increase in model performance on the training data. The algorithm was stopped once 50 decision tree additions occurred without an increase in the AUC on the test data. The final model was the model with all decision trees before the AUC on test data decreased. The entire process of sampling and model building was repeated 1000 times in order to account for sampling biases, and the mean and 95% confidence interval (CI) values of the model performance metrics (accuracy, sensitivity, and specificity) were reported [33]. Before model input, we performed a correlation test on all features (Figure S1).

### 2.5. Importance of Features for Classification

An importance matrix from the XGBoost model was generated that quantifies the contribution of each feature toward classification into diet, DP length, or lactation week and provides their importance based on the relative measure of gain. Gain is the relative contribution of the corresponding feature to the model, calculated by taking each feature’s contribution across all trees in the model. A higher value of gain when compared with another feature implies that it is more important for classifying the dairy cows [32].

### 2.6. Software and Data

All calculations and plots were performed in R (version 4.2.1). The “xgboost” package (version 1.7.11.1) [34] was used to implement XGBoost model. The “ggplot2” package (version 4.0.0) [35] was used for visualization. The ”corrplot” package (version 0.95) [36] was used for correlation analysis.

## 3. Results

### 3.1. Diet Classification

**Figure 2:**
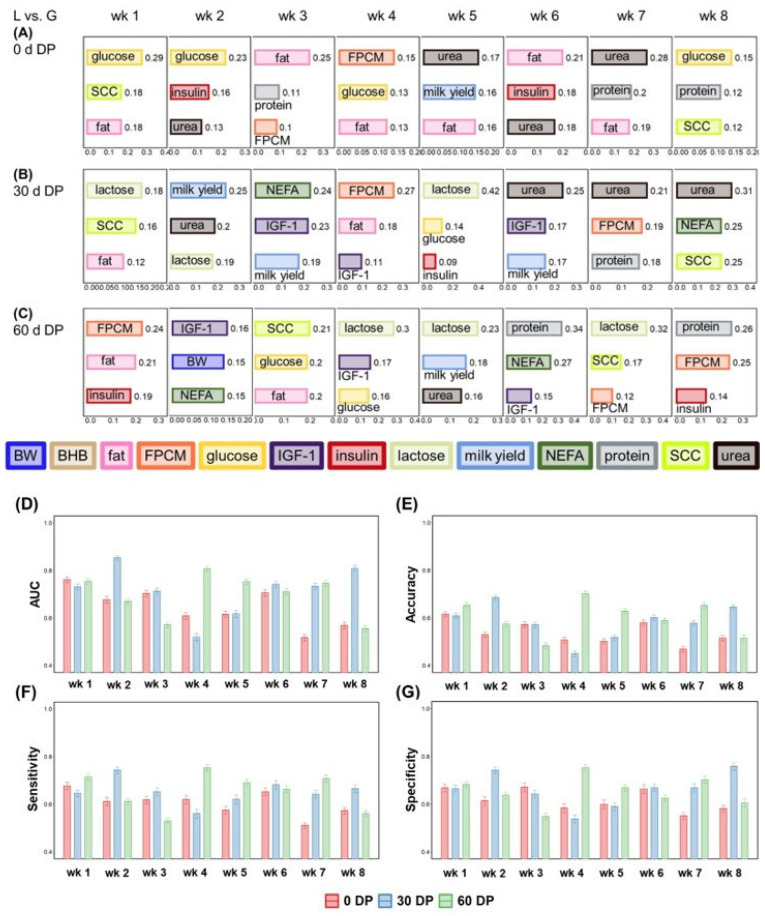
The top 3 features based on gain value to classify cows with a lipogenic diet versus (vs.) a glucogenic diet for (**A**) 0 d DP, (**B**) 30 d DP, or (**C**) 60 d DP from week (wk) 1 to wk 8 using XGBoost algorithm. For the ranking of all features, see Figure S2. (**D**) Mean area under the curve (AUC) of the receiver operating characteristic (ROC), (**E**) mean accuracy, (**F**) mean sensitivity, and (**G**) mean specificity to classify cows with a lipogenic diet vs. a glucogenic diet for a 0 d, 30 d, or 60 d dry period (DP) lengths from wk 1 to wk 8. For specific values, see Table S1. Error bar: 95% confidence interval. Milk fat, milk protein, and milk lactose are expressed in (%). Abbreviations: BW = body weight; DP = dry period; FPCM = fat- and protein-corrected milk production; NEFA = non-esterified fatty acids.

Cows were classified per week and per DP length into either a lipogenic or glucogenic diet based on plasma and milk variables and BW (Figure 2). The top three features, based on gain value in the classification model, are presented in Figure 2A–C. In week 1 and week 2 of 0 d DP, the most important feature to distinguish the lipogenic diet from the glucogenic diet was glucose in plasma. Insulin made an important contribution to classifying cows into diets during early and later lactation weeks. Plasma NEFA was in none of the weeks as one of the important features to classify cows into diets after a 0 d DP, but NEFA was the most important feature in week 3 and week 8 for 30 d DP, and week 2 and week 6 for 60 d DP.

Milk variables were present as the primary features (in 22 of the 24 weeks) to classify diet where milk fat content (in 10 out of the 24 weeks), while milk protein (in 6 of the 24 weeks), milk lactose (in 6 out of the 24 weeks), milk yield (in 5 of the 24 weeks), FPCM yield (in 7 of the 24 weeks), and SCC (in 6 out of the 24 weeks) contributed to a lesser extent. Milk fat content was the most important feature to classify cows into diets than other milk variables.

The performance metrics of the model classifying cows into a lipogenic or glucogenic diet are shown in Figure 2D–G. Overall, model performance to classify cows into diets performed better after a 30 d DP or 60 d DP, and less after a 0 d DP. Comparing the weekly diet classifications, the three DP lengths showed different trends. Classification into diet was best in week 1 for 0 d DP, week 2 for the 30 DP, and week 4 for the 60 DP, based on AUC, accuracy, sensitivity, and specificity values.

### 3.2. Dry Period Length Classification

**Figure 3:**
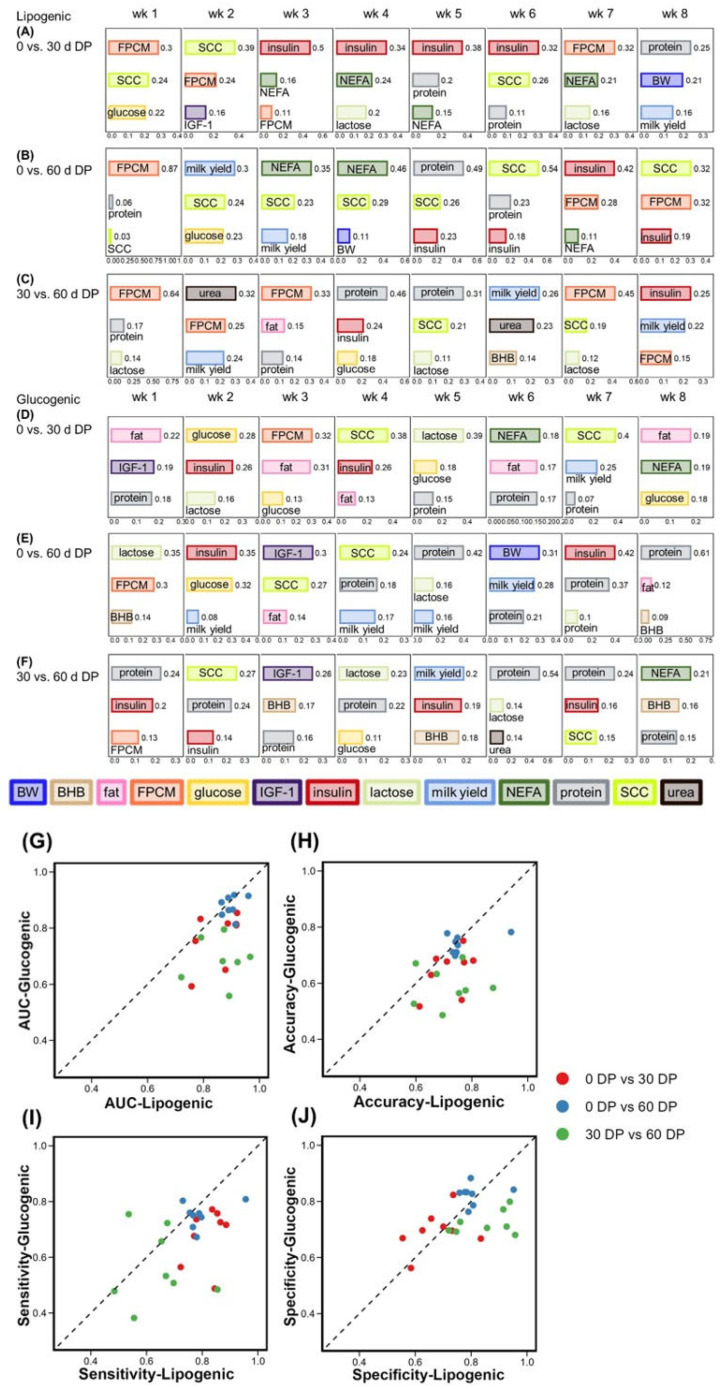
The top 3 features based on gain value to classify (**A**) 0 d DP versus (vs.) 30 d DP, (**B**) 0 d DP vs. 60 d DP, (**C**) 30 DP vs. 60 DP for cows fed the lipogenic diet from week 1 to week 8. For the ranking of all features, see Figure S3. The top 3 features based on gain value to classify (**D**) 0 d DP vs. 30 d DP, (**E**) 0 d DP vs. 60 d DP, and (**F**) 30 DP vs. 60 DP for cows fed the glucogenic diet from week 1 to week 8. For the ranking of all features, see Figure S4. Each point indicates the comparison of 2 DP from week 1 to week 8 and depicts (**G**) the mean area under the curve (AUC) of the receiver operating characteristic (ROC), (**H**) mean accuracy, (**I**) mean sensitivity, and (**J**) mean specificity for cows fed either the lipogenic or glucogenic diet. For actual values, see Tables S2 and S3. Milk fat, milk protein, and milk lactose are expressed as (%). Abbreviations: BW = body weight; DP = dry period; FPCM = fat- and protein-corrected milk production; NEFA = non-esterified fatty acids.

Cows were classified per lactation week and per diet into either 0 d DP, 30 d DP, or 60 d DP based on plasma and milk variables and BW. The top three features, based on the gain value in the classification model, are presented in Figure 3A–F.

For cows fed the lipogenic diet, plasma insulin was present as one of the important features in 10 of 24 weeks to classify cows into DP lengths. Specifically, insulin was the primary feature in 4 of the 8 weeks used to distinguish 0 d DP cows from 30 d DP cows during week 3 to week 6. Plasma glucose (in 3 of 24 weeks) and IGF-1 (in 1 of 24 weeks) were not as important as insulin to classify DP lengths. Plasma NEFA (in 7 of 24 weeks) was present in the key features to classify cows into DP lengths. Milk variables were important for the classification of cows into DP lengths when fed a lipogenic diet. Milk SCC (in 12 of 24 weeks) and milk FPCM (in 12 of the 24 weeks) were important to classify cows into DP lengths.

For cows fed the glucogenic diet, the most important features contributing to the DP length classification were quite different from cows fed the lipogenic diet. For cows fed a glucogenic diet, mainly milk protein content (in 16 of 24 weeks) was important to classify DP length. In addition, milk fat content (in 7 of 16 weeks) was an important feature to classify 0 d DP versus (vs.) 30 d DP (Figure 3D) and 0 d DP vs. 60 d DP (Figure 3 E).

Overall, the discrimination among 0 d DP vs. 30 d DP, 0 d DP vs. 60 d DP, and 30 d DP vs. 60 d DP showed great differences. For both diets, the classification of 0 d DP vs. 60 d DP was stable and had higher values (AUC > 0.8 and specificity > 0.75) than the other two classifications. For both the glucogenic and lipogenic diets, discrimination of 0 d DP vs. 60 d DP was the best, followed by the classification of 0 d DP vs. 30 d DP, and the classification of 30 d DP vs. 60 d DP was the worst. In the classification of 30 d DP vs. 60 d DP, AUC, accuracy, sensitivity, and specificity values were all low from week 1 to week 5. In addition, all three classifications showed the highest AUC value in week 1, which indicated that differences between DP lengths were most obvious in week 1. This indicated that contrasts between DP lengths were clearly reflected in the early lactation week, and the effects would gradually weaken over time.

### 3.3. Lactation Week Classification

**Figure 4:**
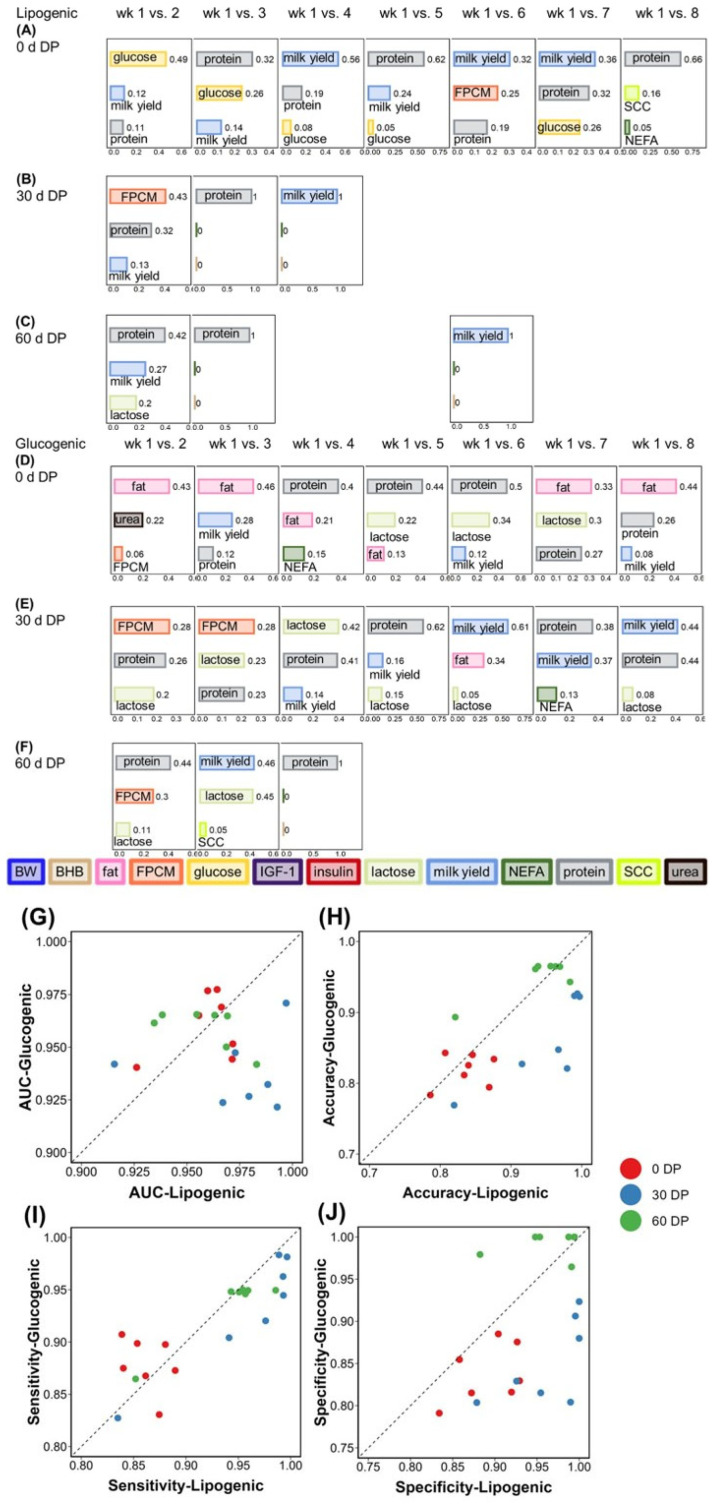
The top 3 features based on gain value used to classify lactation weeks (week 1 vs. week 2, …8) for cows fed the lipogenic diet with(**A**) 0 d DP, (**B**) 30 d DP, or (**C**) 60 d DP, and for cows fed the glucogenic diet with (**D**) 0 d DP, (**E**) 30 d DP, or (**F**) 60 d DP. For the ranking of all features, see Figure S5 (lipogenic diet) and Figure S6 (glucogenic diet). The top 3 features to classify lactation week 1 vs. week 5, …8 for cows fed the lipogenic diet and a 30 d DP were the same as those for week 1 vs. week 4. The importance of all features to classify lactation week 1 vs. week 4 and 5 for cows fed the lipogenic diet and a 60 d DP was the same as that for week 1 vs. week 3. The top 3 features to classify lactation week 7 and 8 vs. week 1 for cows fed the lipogenic diet and a 60 d DP were the same as those for week 1 vs. week 6. The top 3 features to classify lactation week 1 vs. week 2, …8 for cows fed the glucogenic diet and a 60 d DP are the same as those for week 1 vs. week 4. Each point indicates the comparison of lactation week 1 vs. week 2, …8 and depicts (**G**) the mean area under the curve (AUC) of the receiver operating characteristic (ROC), (**H**) mean accuracy, (**I**) mean sensitivity, and (**J**) mean specificity for cows fed either the lipogenic or glucogenic diet. For actual values, see Tables S4 and S5. The units of milk fat, milk protein, and milk lactose are expressed as (%). Abbreviations: BW = body weight; DP = dry period; FPCM = fat- and protein-corrected milk production; NEFA = non-esterified fatty acids.

Cows were classified per diet and per DP length into two lactation weeks (week 1 vs. week 2, 3 …or 8) based on plasma and milk variables and BW. The top three features, based on gain values in the classification model, are presented in Figure 4A–F. For cows fed the lipogenic diet, the most important feature to classify lactation week was milk yield (in 11 of 21 week comparisons), milk protein content (in 8 of 21 weeks), plasma glucose (in 1 of 21 weeks), or FPCM (in 1 of 21 weeks). This may be related to the dynamic changes in the lactation curve during early lactation. For cows fed the glucogenic diet, the top feature to classify lactation week was milk protein content (in 11 of 21 weeks), followed by milk fat content (in 4 of 21 weeks), milk yield (in 3 of 21 weeks), FPCM (in 2 of 21 weeks), and milk lactose content (in 1 of 21 weeks). This is consistent with what we observed in the DP length classification; milk protein content was more important compared with other milk variables for cows fed a glucogenic diet.

For 0 d DP cows fed a lipogenic diet, the most important feature to classify week 1 vs. 2 was plasma glucose concentration. Subsequently, the feature ranking for glucose to classify lactation weeks gradually decreased from week 2 to week 5, and the ranking of milk protein and milk yield increased from week 3 to week 8 vs. week 1. Milk fat percentage was the most important feature to classify week 1 vs. week 2 for the glucogenic diet. Similarly, the ranking of milk fat percentage gradually decreased from week 2 to week 5 vs. week 1.

Lactation week could be well classified in cows fed the lipogenic diet across all three DP lengths, with similar and high AUC values (AUC > 0.9). The three DP lengths had different accuracy, sensitivity, and specificity for classifying lactation week in cows fed the lipogenic diet. The classification accuracy, sensitivity, and specificity of 0 d DP from week 3 to week 8 compared with week 1 were lower than those of 30 d DP and 60 d DP.

## 4. Discussion

In this study, we utilized a machine learning approach to retrospectively classify cows into diet, DP lengths, and lactation week, and provide the feature importance for this classification. The use of plasma and milk variables and the XGBoost algorithm enables the classification of cows into different diets, DP lengths, and lactation weeks to varying degrees. Classification performance of lactation week was the best, showing that AUC is greater than 0.9 and accuracy, sensitivity, and specificity are greater than 0.75, independent of diet or DP length.

The metabolic state of dairy cows changes with the week of lactation, beginning with the occurrence of NEB after calving, which then gradually alleviates and eventually disappears [37]. Followed by the classification of 0 d DP length and 60 d DP length, the performance of AUC is greater than 0.8, and accuracy, sensitivity, and specificity are greater than 0.6, independent of diet or lactation week. Cows with a 0 d DP length avoid severe NEB by reducing milk production in early lactation [9], while cows with a conventional DP of 60 d usually face NEB [38]. This contrast in NEB between 0 d and 60 d DP could clarify when using plasma and milk variables for classification. The performance of the model to classify diets fluctuated greatly across lactation weeks, with mean AUC ranged from 0.85 to 0.52. This illustrates that the effect of dietary strategy on lactation of dairy cows cannot be explained using plasma and milk variables in a very stable manner. Additional variables, including, for example, metabolomics profiles, could contribute to the performance of models to classify cows into diets.

In the current study, plasma glucose played an important role in classification between diets, which might be related to the dietary contrast in the availability of glucogenic nutrients, either at the rumen level in the form of propionate or at the intestinal level in the form of glucose [39]. In an earlier study, a glucogenic diet resulted in a greater plasma insulin concentration in early lactation compared with a lipogenic diet, but did not affect plasma glucose concentration [12]. In contrast, a lipogenic diet in early lactation led to an increase in the concentration of NEFA in the plasma [12], which can inhibit insulin signaling and insulin receptor expression [40]. A more lipogenic diet resulted in a strong increase in milk fat [41,42], possibly corroborating the significant role of milk fat as an important features to discriminate between lipogenic and glucogenic diets.

Remarkably, BHB was never listed among the most important features for diet classification, even though earlier studies reported lower BHB in early lactation for cows fed a more glucogenic diet compared with cows fed a more lipogenic diet [12,43]. This might be related to serum glucose, which is negatively correlated with BHB, and NEFA, which is positively correlated with BHB (Figure S1), had a higher gain value in the classification model. Plasma urea concentration was an important feature for diet classification in 9 of 24 week comparisons. As urea is a product of protein and nitrogen metabolism [44], its concentration indicates that the catabolic status of dairy cows in early lactation not only leads to the loss of body fat but may also lead to the loss of body protein [45]. However, previous analyses have not found that lipogenic or glucogenic diets affect protein balance in dairy cows [46].

In a previous study, cows with a 0 d DP had a lower plasma NEFA concentration post-calving compared with cows with a 30 d or 60 d DP, which is associated with their improved energy balance and lower milk yield [11]. Consistently, plasma insulin concentration was higher in cows with a 0 d DP than in cows with a 30 d DP cow [16]. This is consistent with our study, that is, plasma insulin and glucose are important indicators for distinguishing 0 d DP from 30 d DP and 60 d DP (Figure 3). Elevated plasma insulin inhibits the release of fatty acids from adipose tissue, resulting in a decrease in NEFA in plasma [47]. Moreover, shorter DP resulted in decreased plasma NEFA concentrations in early lactation than traditional dry periods [48]. Although insulin has a clear discriminating role in this study, practical application to use insulin as a discriminating feature in large-scale or on-farm studies should be re-evaluated, related to the high cost and labor-intensive nature. This would limit the applicability of insulin as a feature in on-farm decision support tools, in contrast to glucose as a discriminating feature and being available also in cow-side tests [49]. Attempts have been made to replace plasma glucose measurements with interstitial glucose measurements collected via sensors, but results have been discouraging [50]. Preliminary studies have shown the potential to detect glucose from artificial interstitial fluid using a wearable device [51]. However, it seems that substantial work is still needed before insulin and glucose could be used as near-infrared milk sensors rather than plasma assays as routine on-farm practice.

Milk SCC, FPCM, and protein were also important in classifying cows into DP lengths. Omitting the dry period increased milk SCC in the subsequent lactation compared with a short or conventional dry period [17,52]. Milk from cows after a 0 d DP had a greater fraction of glycosylated κ-casein compared with cows with 30 d DP or 60 d DP, which is considered to be a marker of an increased proportion of unrepaired mammary epithelial cells [53]. This has been attributed either to the omission of dry cow antibiotics or to the accompanying reduction in milk yield and its low dilution effect [17]. While omitting or shortening the DP resulted in a decreased milk yield [52] or FPCM [11], another study reported no overall effect on milk production during the first 100 days of lactation [54].

The classification of lactation week was quite variable across DP lengths and between diets. Classification of cows into weeks was more accurate when cows were fed a lipogenic diet, compared with when cows were fed a glucogenic diet. Cows fed the lipogenic diet had a more pronounced NEB in early lactation due to more body fat mobilization and higher milk fat yield, resulting in a more variable pattern across lactation weeks. Moreover, the NEB in the early stage of lactation is more severe for cows fed the lipogenic diet but will gradually disappear as lactation progresses. This could potentially explain the lower classification efficacy among weeks for cows fed the glucogenic diet compared with cows fed the lipogenic diet. In line with this, cows fed the glucogenic diet had a less severe nadir in energy balance and a more stable variable pattern across lactation weeks [16]. This could clarify the difference in classification between the two diet groups [11,55]. When lipogenic nutrients were less available in the glucogenic diet, milk fat was more important in the classification of lactation week. When glucogenic nutrients were less available in the lipogenic diet, plasma glucose was more important for the classification in week 2 vs. week 1. This might be related to altered hepatic gluconeogenesis [56] or reduced insulin sensitivity [57], as earlier indicated after rumen-protected glucose supplementation.

To conclude, we comment on possible limitations of this study. We allowed a 40% missing data that could be imputed. To assess the effect of imputation on the results, we built classification models on different imputed datasets, which indicated that the results for classification quality did not change, as well as the most important metabolites’ contribution to classifications, even if the upper threshold for missingness was set to 40% which may seem a rather high threshold. The good replicability over different imputed solutions seems to be in line with observations that unbiased estimation can be obtained even in the presence of 90% missing data [58].

This study presented a relatively small sample size of 95 animals. However, the cross-validation strategy employed limits the possibility of overfitting for what is possible in this type of study and analysis. Nevertheless, increasing the sample size could improve the robustness of the findings and enhance the generalizability of the results. This generalizability could be further assessed and confirmed using an external cohort of animals to act as an independent validation cohort, either by replicating the analysis on new samples or transferring model parameters to the analysis of new data to assess generalizability, upon deciding at which level generalizability is sought: temporal, geographical, and domain generalizability [59,60]. In the context of this study and field of application, it would probably be meaningful to assess the model at the geographical level, with new data from different regions collected with similar experimental designs.

## 5. Conclusions

In this study, cows were classified into diet, DP length, and lactation week using BW and plasma and milk variables using the XGBoost algorithm. Classification feasibility lactation weeks achieved the highest metrics. Feature importance profiles for distinguishing diets or DP lengths varied across lactation weeks. Key discriminative biomarkers for diet classification were plasma urea concentration and milk fat content. For cows receiving a lipogenic diet, DP length was primarily predicted by plasma insulin, NEFA, milk SCC, and FPCM yield. In contrast, milk protein content emerged as the predominant predictor of DP length for cows fed a glucogenic diet. Classification of lactation week in the lipogenic-diet group relied mainly on milk protein content and milk yield, whereas lactation week for cows fed the glucogenic diet was most effectively differentiated using milk fat content, milk protein content, and milk yield. Overall, the current results imply that machine learning methods using blood and milk biomarkers could support producers, veterinarians, or farm advisors to track causes of health or performance problems and, herewith, facilitate management adjustments for improved cow health and performance. Future research should expand the feature dataset to include sensor-derived data and metabolomic profiles and explore alternative modeling approaches for classifying health status or evaluating a broader range of dietary interventions or management strategies.

## Figures and Tables

**Table 1:** Ingredient and calculated chemical composition (g/kg of DM, unless otherwise stated) of postpartum rations. Adjusted from Van Knegsel et al. [11].

Composition	Glucogenic	Lipogenic
Ingredient		
Grass silage	338	338
Corn silage	227	227
Soybean meal	46	46
Rapeseed meal	36	36
Rapeseed straw	10	10
Wheat straw	5	5
Concentrate	338	338
Chemical composition		
DM (g/kg of product)	561	566
CP	167	169
Crude fat	31	37
NDF	318	389
ADF	182	224
Starch	215	106
Sugars	82	85
Ash	76	80
DVE	87	84
OEB	17	17
NE (MJ/kg of DM)	6.55	6.52

## Data Availability

The data are available at https://github.com/esaccenti/CowLipoGlucoDiet (accessed on 22 February 2023).

## Supplementary Materials

The following supporting information can be downloaded at: https://www.mdpi.com/article/10.3390/metabo15110698/s1, Figure S1. Pearson correlation analysis of all input features. Figure S2. Feature contribution to classify cows into lipogenic diet or glucogenic diet for cows with 0 d dry period length (DP), 30 d DP, or 60 d DP through week (wk) 1. Figure S3. Feature contribution based on gain value to classify 0 d DP versus (vs.) 30 d DP, 0 d DP vs. 60 d DP, 30 DP vs. 60 DP for cows fed with lipogenic diet and through week (wk) 1 to wk 8. Figure S4. Feature contribution based on gain value to classify 0 d DP versus (vs.) 30 d DP, 0 d DP vs. 60 d DP, 30 DP vs. 60 DP for cows fed with glucogenic diet and through week (wk) 1 to wk 8 using plasma and milk variables. Figure S5. Feature contribution based on gain value to classify lactation weeks (wk 1 vs. wk 2, …8) for cows fed the lipogenic diet and 0 d DP, 30 d DP, or 60 d DP using plasma and milk. Figure S6. Feature contribution based on gain value to classify lactation weeks (wk 1 vs. wk 2, …8) for cows fed the lipogenic diet for cows fed the glucogenic diet and 0 d DP, 30 d DP, or 60 d DP using plasma and milk variables. Table S1. Model performance to classify cows into lipogenic diet or glucogenic diet for cows with 0 d dry period length (DP), 30 d DP, or 60 d DP through week (wk)1 to wk 8. Table S2. Model performance to classify 0 d DP versus (vs.) 30 d DP, 0 d DP vs. 60 d DP, 30 DP vs. 60 DP for cows fed with lipogenic diet and through week (wk)1 to wk 8. Table S3. Model performance to classify cows 0 d DP versus (vs.) 30 d DP, 0 d DP vs. 60 d DP, 30 DP vs. 60 DP for cows fed with glucogenic diet and through week (wk)1 to wk 8. Table S4. Model performance to classify lactation week (wk 1 vs. wk 2, …8) for cows fed the lipogenic diet. Table S5. Model performance to classify lactation week (wk 1 vs. wk 2, …8) for cows fed the glucogenic diet.
