# Supplementary material for: Plasma and Milk Variables Classify Diet, Dry Period Length, and Lactation Week of Dairy Cows Using a Machine Learning Approach
Source: Metabolites. 2025 Oct 28;15(11):698. doi: 10.3390/metabo15110698 (PMC12654569; doi:10.3390/metabo15110698)
Supplement: Supplementary file 1 [file metabolites-15-00698-s001.zip › metabolites-3708988-supplementary.pdf]

metabolites

MDPI

Supplementary Material

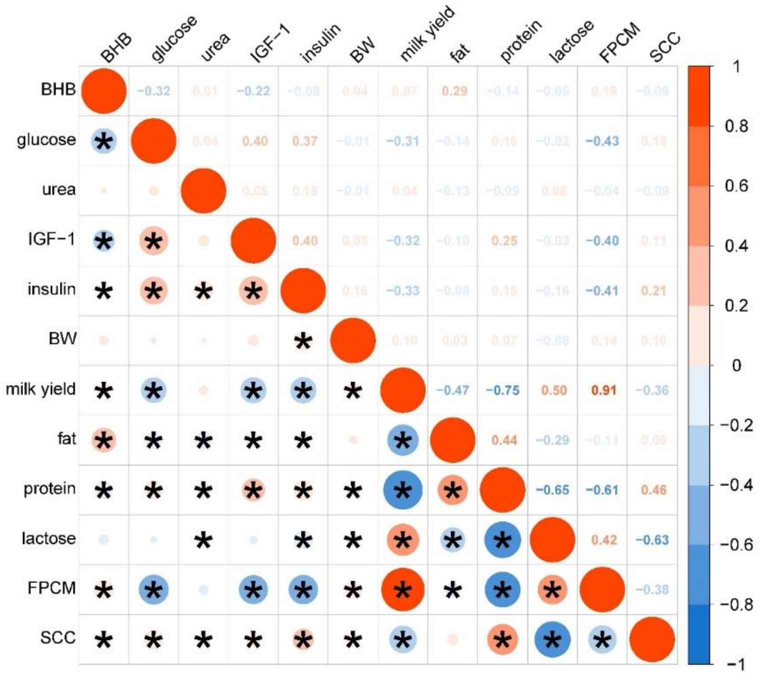

Figure S1. Pearson correlation analysis of all input features. Abbreviations: BW = body weight; DP = dry period; FPCM = fat- and protein- corrected milk production; NEFA = non-esterified fatty acids.

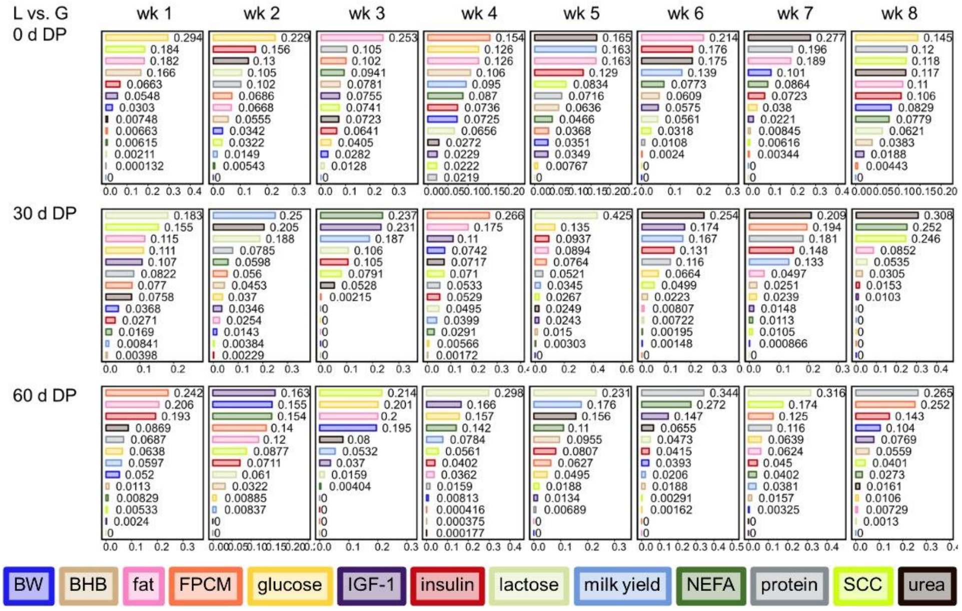

Figure S2. Feature contribution to classify cows into lipogenic diet or glucogenic diet for cows with 0 d dry period length (DP), 30 d DP, or 60 d DP through week (wk) 1 to wk 8 using plasma and milk variables and a XGBoost machine learning algorithm. Values indicate the gain value and features are in ranking order.

The unit of milk fat, milk protein, and milk lactose is content (%).

Abbreviations: DP = dry period; FPCM = fat- and protein- corrected milk production; NEFA = non-esterified fatty acids; IGF-1 = Insulin-like growth factor 1; BHB = Beta-hydroxybutyrate; SCC = Somatic cell count in milk; BW = Body weight.

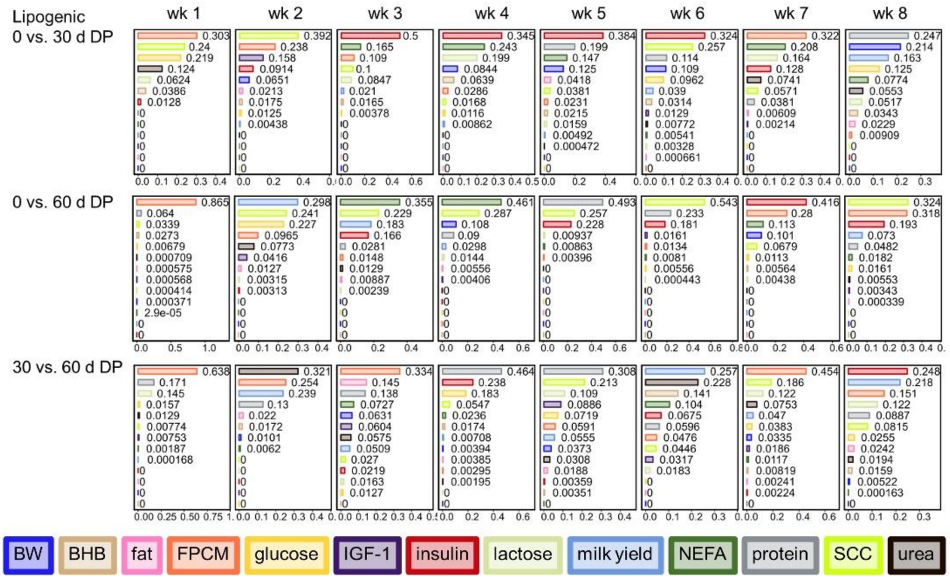

Figure S3. Feature contribution based on gain value to classify 0 d DP versus (vs.) 30 d DP, 0 d DP vs. 60 d DP, 30 DP vs. 60 DP for cows fed with lipogenic diet and through week (wk) 1 to wk 8 using plasma and milk variables and a XGBoost machine learning algorithm. Values indicate the gain value and features are in ranking order.

The unit of milk fat, milk protein, and milk lactose is content (%).

Abbreviations: DP = dry period; FPCM = fat- and protein- corrected milk production; NEFA = non-esterified fatty acids; IGF-1 = Insulin-like growth factor 1; BHB = Beta-hydroxybutyrate; SCC = Somatic cell count in milk; BW = Body weight.

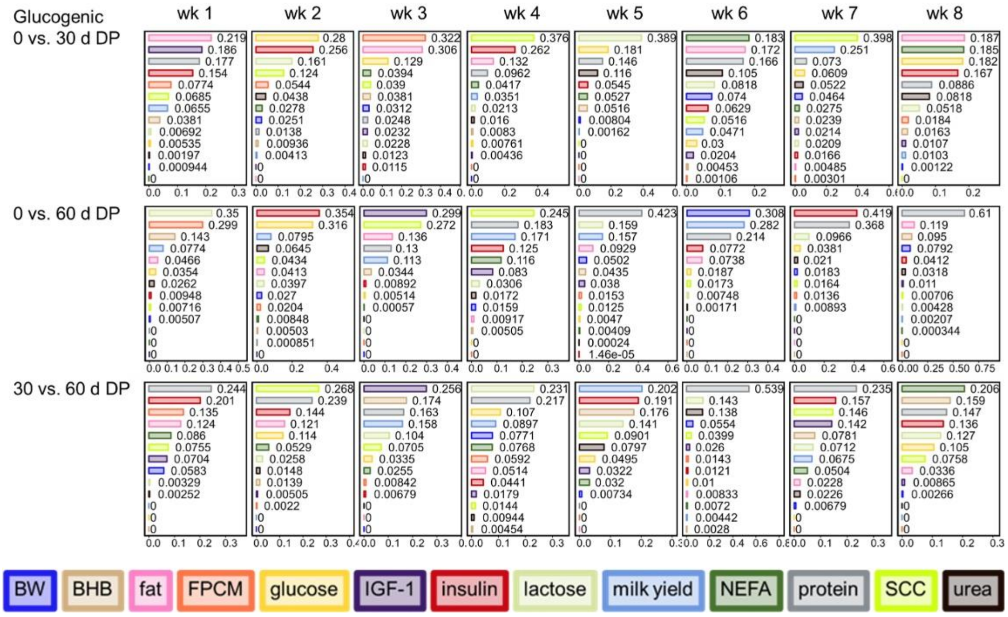

Figure S4. Feature contribution based on gain value to classify 0 d DP versus (vs.) 30 d DP, 0 d DP vs. 60 d DP, 30 DP vs. 60 DP for cows fed with glucogenic diet and through week (wk) 1 to wk 8 using plasma and milk variables and a XGBoost machine learning algorithm. Values indicate the gain value and features are in ranking order.

The unit of milk fat, milk protein, and milk lactose is content (%).

Abbreviations: DP = dry period; FPCM = fat- and protein- corrected milk production; NEFA = non-esterified fatty acids; IGF-1 = Insulin-like growth factor 1; BHB = Beta-hydroxybutyrate; SCC = Somatic cell count in milk; BW = Body weight.

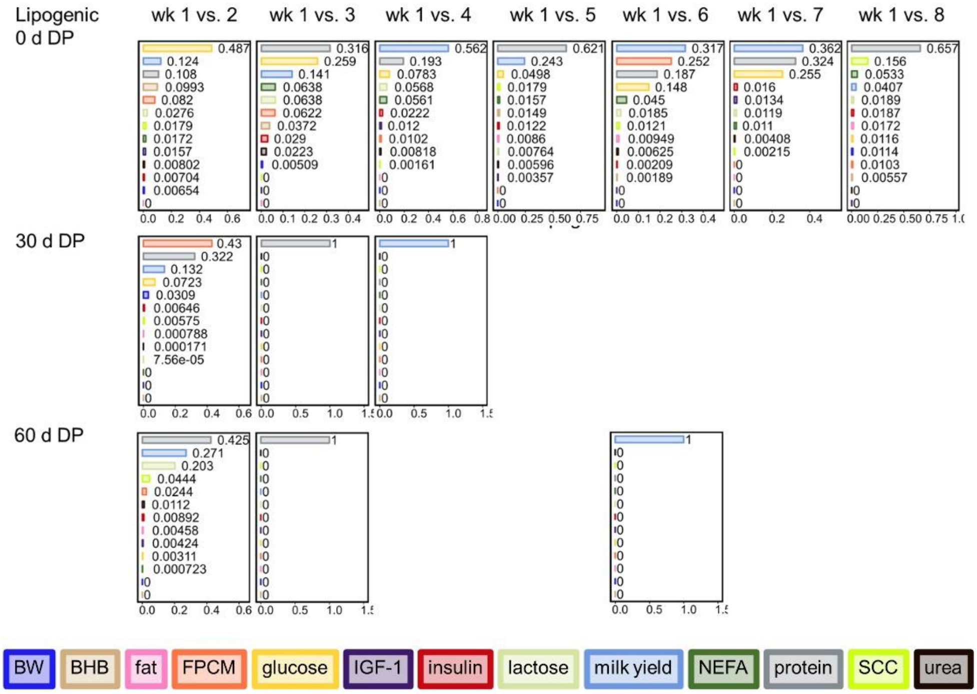

Figure S5. Feature contribution based on gain value to classify lactation weeks (wk 1 vs. wk 2, …8) for cows fed the lipogenic diet and 0 d DP, 30 d DP, or 60 d DP using plasma and milk variables and a XGBoost machine learning algorithm. Values indicate the gain value and features are in ranking order.

The unit of milk fat, milk protein, and milk lactose is content (%).

Abbreviations: DP = dry period; FPCM = fat- and protein- corrected milk production; NEFA = non-esterified fatty acids; IGF-1 = Insulin-like growth factor 1; BHB = Beta-hydroxybutyrate; SCC = Somatic cell count in milk; BW = Body weight.

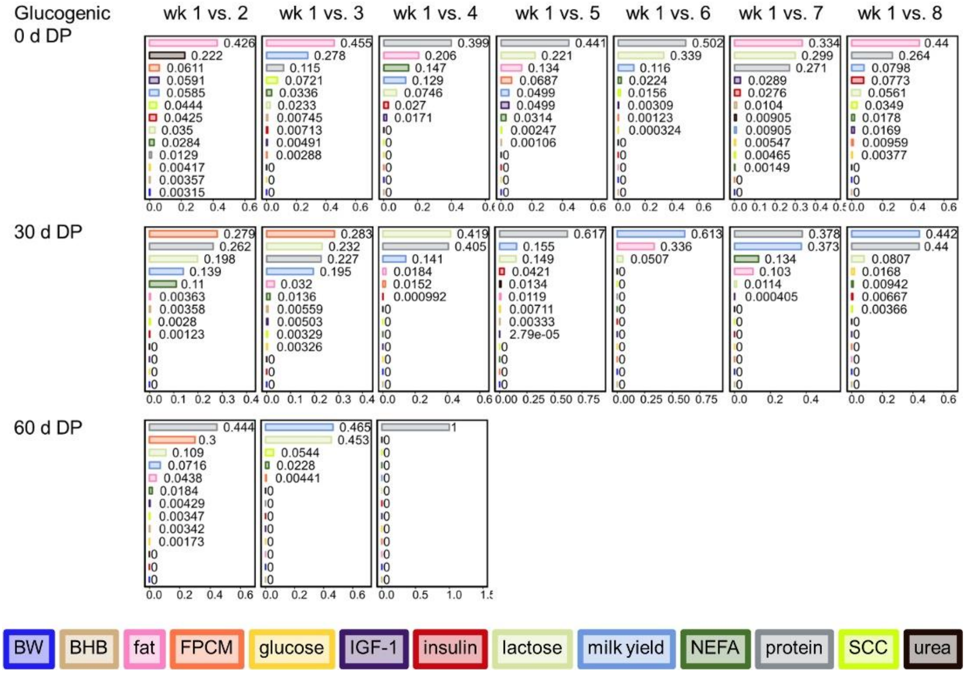

Figure S6 Feature contribution based on gain value to classify lactation weeks (wk 1 vs. wk 2, …8) for cows fed the lipogenic diet for cows fed the glucogenic diet and 0 d DP, 30 d DP, or 60 d DP using plasma and milk variables and a XGBoost machine learning algorithm. Values indicate the gain value and features are in ranking order.

The unit of milk fat, milk protein, and milk lactose is content (%).

Abbreviations: DP = dry period; FPCM = fat- and protein- corrected milk production; NEFA = non-esterified fatty acids; IGF-1 = Insulin-like growth factor 1; BHB = Beta-hydroxybutyrate; SCC = Somatic cell count in milk; BW = Body weight.

Table S1. Model performance (AUC, accuracy, sensitivity, and specificity) to classify cows into lipogenic diet or glucogenic diet for cows with 0 d dry period length (DP), 30 d DP, or 60 d DP through week (wk)1 to wk 8.

lactation wk	DP	AUC		Accuracy		Sensitivity		Specificity	
		Mean	95% Confidence Interval	Mean	95% Confidence Interval	Mean	95% Confidence Interval	Mean	95% Confidence Interval
week1	0 DP	0.761	0.748 - 0.773	0.615	0.605 -0.626	0.676	0.66-0.691	0.667	0.652-0.683
wk2	0 DP	0.676	0.662 - 0.690	0.529	0.517 -0.54	0.611	0.594-0.628	0.613	0.595-0.63
wk3	0 DP	0.703	0.688 - 0.717	0.572	0.56 -0.583	0.618	0.603-0.634	0.670	0.652-0.688
wk4	0 DP	0.608	0.594 - 0.622	0.506	0.494 -0.518	0.619	0.601-0.636	0.583	0.564-0.601
wk5	0 DP	0.614	0.600 - 0.629	0.501	0.489 -0.512	0.574	0.558-0.589	0.599	0.58-0.617
wk6	0 DP	0.705	0.691 - 0.719	0.579	0.566 -0.592	0.651	0.635-0.667	0.663	0.644-0.682
wk7	0 DP	0.516	0.503 - 0.529	0.469	0.457 -0.48	0.510	0.499-0.522	0.550	0.535-0.565
wk8	0 DP	0.568	0.555 - 0.582	0.515	0.504 -0.527	0.572	0.558-0.586	0.580	0.567-0.593
wk1	30 DP	0.730	0.718 - 0.742	0.608	0.597 -0.619	0.646	0.633-0.659	0.664	0.65-0.678
wk2	30 DP	0.852	0.842 - 0.862	0.684	0.674 -0.695	0.743	0.73-0.757	0.741	0.727-0.754
wk3	30 DP	0.712	0.698 - 0.726	0.570	0.558 -0.582	0.652	0.634-0.669	0.642	0.626-0.659
wk4	30 DP	0.519	0.504 - 0.534	0.450	0.438 -0.461	0.559	0.54-0.578	0.537	0.52-0.554
wk5	30 DP	0.617	0.602 - 0.632	0.517	0.505 -0.529	0.620	0.601-0.638	0.589	0.573-0.605
wk6	30 DP	0.741	0.727 - 0.755	0.601	0.589 -0.612	0.681	0.663-0.699	0.669	0.653-0.684
wk7	30 DP	0.732	0.719 - 0.746	0.577	0.566 -0.589	0.642	0.624-0.659	0.667	0.65-0.684
wk8	30 DP	0.808	0.795 - 0.821	0.645	0.634 -0.656	0.664	0.648-0.68	0.757	0.741-0.774
wk1	60 DP	0.752	0.741 - 0.763	0.653	0.642 -0.664	0.713	0.7-0.727	0.682	0.669-0.694
wk2	60 DP	0.669	0.657 - 0.681	0.573	0.563 -0.584	0.612	0.599-0.624	0.636	0.622-0.65
wk3	60 DP	0.571	0.558 - 0.583	0.482	0.471 -0.493	0.529	0.516-0.541	0.547	0.533-0.561
wk4	60 DP	0.805	0.794 - 0.817	0.701	0.69 -0.712	0.752	0.738-0.765	0.753	0.739-0.766
wk5	60 DP	0.751	0.739 - 0.763	0.628	0.617 -0.638	0.690	0.676-0.704	0.668	0.655-0.681
wk6	60 DP	0.709	0.697 - 0.721	0.588	0.576 -0.599	0.662	0.648-0.677	0.625	0.611-0.638
wk7	60 DP	0.745	0.733 - 0.757	0.652	0.639 -0.665	0.707	0.692-0.722	0.702	0.687-0.716
wk8	60 DP	0.555	0.541 - 0.569	0.515	0.502 -0.528	0.559	0.546-0.573	0.604	0.587-0.62

Abbreviation: AUC = area under curve of the receiver operating characteristic (ROC).

Table S2. Model performance (AUC, accuracy, sensitivity, and specificity) to classify 0 d DP versus (vs.) 30 d DP, 0 d DP vs. 60 d DP, 30 DP vs. 60 DP for cows fed with lipogenic diet and through week (wk)1 to wk 8.

Comparison	lactation wk	AUC		Accuracy		Sensitivity		Specificity	
		Mean	95% Confidence Interval	Mean	95% Confidence Interval	Mean	95% Confidence Interval	Mean	95% Confidence Interval
0 DP vs. 30 DP	wk1	0.920	0.915-0.926	0.769	0.762-0.776	0.836	0.828-0.844	0.736	0.724-0.748
0 DP vs. 30 DP	wk 2	0.917	0.911-0.924	0.805	0.797-0.813	0.854	0.847-0.862	0.835	0.822-0.848
0 DP vs. 30 DP	wk 3	0.917	0.911-0.923	0.772	0.764-0.781	0.886	0.878-0.894	0.701	0.689-0.712
0 DP vs. 30 DP	wk 4	0.887	0.88-0.894	0.711	0.702-0.72	0.865	0.856-0.874	0.626	0.614-0.637
0 DP vs. 30 DP	wk 5	0.771	0.762-0.78	0.654	0.646-0.663	0.771	0.762-0.78	0.555	0.546-0.565
0 DP vs. 30 DP	wk 6	0.879	0.872-0.885	0.764	0.756-0.771	0.845	0.837-0.853	0.732	0.719-0.744
0 DP vs. 30 DP	wk 7	0.757	0.746-0.769	0.613	0.603-0.623	0.723	0.71-0.735	0.585	0.572-0.597
0 DP vs. 30 DP	wk 8	0.789	0.779-0.8	0.673	0.663-0.682	0.779	0.768-0.79	0.657	0.644-0.67
0 DP vs. 60 DP	wk 1	0.961	0.956-0.965	0.940	0.935-0.946	0.956	0.95-0.963	0.952	0.946-0.958
0 DP vs. 60 DP	wk 2	0.890	0.882-0.898	0.742	0.732-0.752	0.789	0.777-0.801	0.759	0.747-0.771
0 DP vs. 60 DP	wk 3	0.889	0.879-0.899	0.750	0.738-0.761	0.797	0.784-0.81	0.778	0.765-0.791

0 DP vs. 60 DP	wk 4	0.910	0.903-0.917	0.748	0.739-0.758	0.767	0.756-0.778	0.804	0.792-0.815
0 DP vs. 60 DP	wk 5	0.905	0.897-0.913	0.745	0.735-0.755	0.780	0.768-0.792	0.799	0.786-0.811
0 DP vs. 60 DP	wk 6	0.914	0.906-0.922	0.740	0.73-0.75	0.767	0.754-0.779	0.807	0.795-0.82
0 DP vs. 60 DP	wk 7	0.866	0.857-0.875	0.732	0.722-0.742	0.756	0.744-0.767	0.790	0.778-0.803
0 DP vs. 60 DP	wk 8	0.865	0.855-0.874	0.712	0.702-0.722	0.730	0.719-0.742	0.784	0.771-0.797
30 DP vs. 60 DP	wk 1	0.967	0.946-0.988	0.876	0.852-0.9	0.855	0.8-0.909	0.938	0.908-0.969
30 DP vs. 60 DP	wk 2	0.892	0.853-0.931	0.695	0.657-0.733	0.555	0.506-0.605	0.958	0.925-0.991
30 DP vs. 60 DP	wk 3	0.922	0.882-0.962	0.754	0.704-0.805	0.697	0.618-0.777	0.857	0.813-0.901
30 DP vs. 60 DP	wk 4	0.869	0.833-0.905	0.778	0.728-0.828	0.670	0.602-0.737	0.927	0.886-0.968
30 DP vs. 60 DP	wk 5	0.721	0.671-0.771	0.593	0.546-0.64	0.485	0.435-0.535	0.747	0.696-0.798
30 DP vs. 60 DP	wk 6	0.874	0.816-0.931	0.767	0.709-0.825	0.675	0.612-0.737	0.915	0.864-0.966
30 DP vs. 60 DP	wk 7	0.791	0.772-0.811	0.674	0.654-0.694	0.654	0.626-0.681	0.761	0.739-0.783
30 DP vs. 60 DP	W8	0.740	0.72-0.759	0.599	0.582-0.616	0.536	0.52-0.552	0.720	0.696-0.744

Abbreviation: AUC = area under curve of the receiver operating characteristic (ROC).

Table S3. Model performance (AUC, accuracy, sensitivity, and specificity) to classify cows 0 d DP versus (vs.) 30 d DP, 0 d DP vs. 60 d DP, 30 DP vs. 60 DP for cows fed with glucogenic diet and through week (wk)1 to wk 8.

Comparison	lactation wk	AUC		Accuracy		Sensitivity		Specificity	
		Mean	95% Confidence Interval	Mean	95% Confidence Interval	Mean	95% Confidence Interval	Mean	95% Confidence Interval
0 DP vs. 30 DP	wk1	0.854	0.847-0.861	0.751	0.743-0.759	0.772	0.764-0.78	0.823	0.81-0.836
0 DP vs. 30 DP	wk2	0.814	0.806-0.823	0.681	0.672-0.69	0.758	0.747-0.768	0.668	0.656-0.679
0 DP vs. 30 DP	wk3	0.810	0.801-0.819	0.675	0.666-0.683	0.716	0.705-0.728	0.710	0.699-0.721
0 DP vs. 30 DP	wk4	0.816	0.807-0.826	0.678	0.668-0.687	0.726	0.713-0.738	0.697	0.686-0.708
0 DP vs. 30 DP	wk5	0.754	0.744-0.764	0.630	0.62-0.639	0.676	0.664-0.689	0.669	0.657-0.681
0 DP vs. 30 DP	wk6	0.652	0.632-0.672	0.540	0.522-0.559	0.488	0.469-0.506	0.696	0.668-0.723
0 DP vs. 30 DP	wk7	0.593	0.582-0.604	0.517	0.507-0.528	0.565	0.552-0.578	0.563	0.549-0.576
0 DP vs. 30 DP	wk8	0.832	0.822-0.842	0.687	0.676-0.699	0.736	0.723-0.75	0.739	0.725-0.753
0 DP vs. 60 DP	wk1	0.915	0.903-0.927	0.782	0.765-0.799	0.808	0.783-0.833	0.842	0.824-0.859
0 DP vs. 60 DP	wk2	0.864	0.845-0.883	0.747	0.723-0.771	0.758	0.727-0.788	0.831	0.809-0.853
0 DP vs. 60 DP	wk3	0.908	0.896-0.92	0.735	0.722-0.749	0.744	0.718-0.769	0.833	0.815-0.851
0 DP vs. 60 DP	wk4	0.918	0.906-0.93	0.762	0.746-0.777	0.751	0.727-0.774	0.827	0.808-0.847
0 DP vs. 60 DP	wk5	0.866	0.847-0.884	0.711	0.695-0.726	0.672	0.65-0.695	0.883	0.863-0.903
0 DP vs. 60 DP	wk6	0.813	0.796-0.83	0.698	0.681-0.716	0.708	0.68-0.737	0.786	0.766-0.807
0 DP vs. 60 DP	wk7	0.848	0.838-0.857	0.711	0.702-0.72	0.760	0.747-0.773	0.763	0.75-0.776
0 DP vs. 60 DP	wk8	0.892	0.883-0.9	0.777	0.768-0.786	0.803	0.791-0.814	0.833	0.822-0.844
30 DP vs. 60 DP	wk1	0.698	0.614-0.783	0.583	0.509-0.658	0.484	0.403-0.566	0.799	0.728-0.869
30 DP vs. 60 DP	wk2	0.559	0.504-0.614	0.486	0.443-0.529	0.382	0.34-0.425	0.681	0.624-0.737
30 DP vs. 60 DP	wk3	0.679	0.656-0.702	0.565	0.547-0.582	0.508	0.492-0.524	0.705	0.68-0.731
30 DP vs. 60 DP	wk4	0.683	0.657-0.709	0.575	0.553-0.596	0.533	0.511-0.555	0.710	0.682-0.738
30 DP vs. 60 DP	wk5	0.625	0.603-0.648	0.527	0.506-0.547	0.479	0.459-0.498	0.692	0.661-0.723
30 DP vs. 60 DP	wk6	0.795	0.786-0.804	0.693	0.685-0.701	0.723	0.711-0.735	0.771	0.759-0.784
30 DP vs. 60 DP	wk7	0.766	0.754-0.778	0.633	0.623-0.644	0.657	0.645-0.669	0.727	0.712-0.742
30 DP vs. 60 DP	wk8	0.786	0.776-0.797	0.671	0.661-0.681	0.755	0.741-0.769	0.697	0.685-0.708

Abbreviation: AUC = area under curve of the receiver operating characteristic (ROC).

Table S4. Model performance (AUC, accuracy, sensitivity, and specificity) to classify lactation week (wk 1 vs. wk 2, …8) for cows fed the lipogenic diet.

Comparison	DP	AUC		Accuracy		Sensitivity		Specificity	
		Mean	95% Confidence Interval	Mean	95% Confidence Interval	Mean	95% Confidence Interval	Mean	95% Confidence Interval
wk1 vs wk2	0 DP	0.926	0.921-0.932	0.786	0.778-0.794	0.840	0.83-0.851	0.834	0.823-0.845
wk1 vs wk3	0 DP	0.972	0.967-0.976	0.834	0.825-0.843	0.880	0.87-0.891	0.872	0.862-0.883
wk1 vs wk4	0 DP	0.971	0.967-0.976	0.869	0.861-0.877	0.874	0.865-0.884	0.929	0.921-0.938
wk1 vs wk5	0 DP	0.956	0.95-0.961	0.846	0.836-0.855	0.861	0.851-0.872	0.904	0.894-0.914
wk1 vs wk6	0 DP	0.960	0.954-0.965	0.876	0.868-0.884	0.890	0.881-0.899	0.926	0.918-0.935
wk1 vs wk7	0 DP	0.964	0.959-0.97	0.840	0.832-0.848	0.839	0.829-0.849	0.920	0.911-0.929
wk1 vs wk8	0 DP	0.966	0.962-0.971	0.807	0.8-0.814	0.853	0.844-0.863	0.858	0.848-0.868
wk1 vs wk2	30 DP	0.973	0.969-0.976	0.820	0.813-0.826	0.835	0.826-0.843	0.879	0.869-0.888
wk1 vs wk3	30 DP	0.916	0.91-0.921	0.916	0.91-0.921	0.941	0.935-0.948	0.926	0.919-0.933
wk1 vs wk4	30 DP	0.967	0.964-0.97	0.967	0.964-0.97	0.993	0.991-0.995	0.954	0.949-0.96
wk1 vs wk5	30 DP	0.979	0.975-0.984	0.979	0.975-0.984	0.976	0.971-0.981	0.990	0.986-0.993
wk1 vs wk6	30 DP	0.997	0.995-0.999	0.997	0.995-0.999	0.997	0.994-0.999	1.000	1-1
wk1 vs wk7	30 DP	0.993	0.986-0.999	0.994	0.988-0.999	0.993	0.986-0.999	1.000	1-1
wk1 vs wk8	30 DP	0.988	0.98-0.997	0.990	0.982-0.997	0.989	0.981-0.997	0.995	0.989-1.002
wk1 vs wk2	60 DP	0.969	0.964-0.973	82.127	0.814-0.829	0.852	0.842-0.862	0.882	0.873-0.892
wk1 vs wk3	60 DP	0.983	0.978-0.988	98.348	0.979-0.988	0.986	0.981-0.99	0.991	0.988-0.994
wk1 vs wk4	60 DP	0.955	0.949-0.961	95.603	0.950-0.962	0.943	0.936-0.95	0.988	0.984-0.991
wk1 vs wk5	60 DP	0.935	0.929-0.94	93.450	0.929-0.94	0.957	0.951-0.963	0.948	0.941-0.955
wk1 vs wk6	60 DP	0.969	0.965-0.973	96.917	0.965-0.973	0.959	0.954-0.965	0.995	0.992-0.997
wk1 vs wk7	60 DP	0.963	0.959-0.967	96.317	0.959-0.967	0.951	0.945-0.957	0.994	0.992-0.996
wk1 vs wk8	60 DP	0.938	0.933-0.943	93.833	0.933-0.943	0.954	0.948-0.96	0.953	0.947-0.959

Abbreviation: AUC = area under curve of the receiver operating characteristic (ROC).

Table S5. Model performance (AUC, accuracy, sensitivity, and specificity) to classify lactation week (wk 1 vs. wk 2, …8) for cows fed the glucogenic diet.

Comparison	DP	AUC		Accuracy		Sensitivity		Specificity	
		Mean	95% Confidence Interval	Mean	95% Confidence Interval	Mean	95% Confidence Interval	Mean	95% Confidence Interval
wk1 vs wk2	0 DP	0.940	0.936-0.945	0.783	0.777-0.79	0.875	0.865-0.885	0.791	0.782-0.8
wk1 vs wk3	0 DP	0.952	0.948-0.955	0.812	0.805-0.818	0.898	0.889-0.906	0.815	0.806-0.825
wk1 vs wk4	0 DP	0.944	0.94-0.949	0.794	0.787-0.802	0.831	0.821-0.84	0.829	0.82-0.839
wk1 vs wk5	0 DP	0.965	0.961-0.969	0.840	0.832-0.848	0.868	0.858-0.877	0.885	0.875-0.895
wk1 vs wk6	0 DP	0.977	0.973-0.981	0.834	0.828-0.84	0.873	0.865-0.881	0.875	0.866-0.885
wk1 vs wk7	0 DP	0.977	0.971-0.983	0.825	0.811-0.84	0.907	0.892-0.923	0.816	0.794-0.838

wk1 vs wk8	0 DP	0.969	0.961-0.977	0.843	0.83-0.856	0.899	0.884-0.913	0.855	0.834-0.875
wk1 vs wk2	30 DP	0.947	0.943-0.951	0.769	0.762-0.776	0.827	0.817-0.838	0.804	0.794-0.813
wk1 vs wk3	30 DP	0.942	0.926-0.958	0.827	0.814-0.841	0.904	0.889-0.919	0.829	0.806-0.852
wk1 vs wk4	30 DP	0.924	0.908-0.94	0.847	0.833-0.862	0.945	0.931-0.958	0.815	0.794-0.836
wk1 vs wk5	30 DP	0.927	0.911-0.942	0.821	0.813-0.829	0.920	0.906-0.935	0.804	0.784-0.824
wk1 vs wk6	30 DP	0.971	0.952-0.989	0.922	0.894-0.951	0.981	0.963-0.999	0.880	0.828-0.932
wk1 vs wk7	30 DP	0.922	0.881-0.963	0.927	0.887-0.966	0.963	0.937-0.988	0.923	0.868-0.978
wk1 vs wk8	30 DP	0.932	0.9-0.965	0.924	0.885-0.962	0.983	0.967-1	0.906	0.849-0.964
wk1 vs wk2	60 DP	0.950	0.945-0.955	0.893	0.886-0.901	0.865	0.855-0.874	0.979	0.974-0.985
wk1 vs wk3	60 DP	0.942	0.937-0.947	0.943	0.938-0.948	0.950	0.943-0.956	0.965	0.959-0.97
wk1 vs wk4	60 DP	0.966	0.961-0.97	0.966	0.961-0.97	0.948	0.942-0.955	1.000	1-1
wk1 vs wk5	60 DP	0.962	0.956-0.967	0.962	0.956-0.967	0.946	0.939-0.953	1.000	1-1
wk1 vs wk6	60 DP	0.965	0.96-0.97	0.965	0.96-0.97	0.949	0.943-0.956	1.000	1-1
wk1 vs wk7	60 DP	0.965	0.961-0.969	0.965	0.961-0.969	0.948	0.941-0.954	1.000	1-1
wk1 vs wk8	60 DP	0.965	0.961-0.97	0.965	0.961-0.97	0.950	0.943-0.957	1.000	1-1

Abbreviation: AUC = area under curve of the receiver operating characteristic (ROC).

Disclaimer/Publisher's Note: The statements, opinions and data contained in all publications are solely those of the individual author(s) and contributor(s) and not of MDPI and/or the editor(s). MDPI and/or the editor(s) disclaim responsibility for any injury to people or property resulting from any ideas, methods, instructions or products referred to in the content.
